# Fatty acids composition and in vivo biochemical effects of *Aleurites moluccana* seed (Candlenut) in obese wistar rats

**DOI:** 10.1186/s13098-022-00847-4

**Published:** 2022-06-08

**Authors:** Matheus Camargos de Britto Rosa, Paula Reis Ribeiro, Viviam de Oliveira Silva, Danubia Aparecida de Carvalho Selvati-Rezende, Tácio Peres da Silva, Fernanda Rezende Souza, Maria das Graças Cardoso, Josilene Nascimento Seixas, Eric Francelino Andrade, Vanessa Pardi, Ramiro Mendonça Murata, Luciano José Pereira

**Affiliations:** 1 Veterinary Medicine Department, Universidade Federal de Lavras, Mail Box 3037, Lavras, Minas Gerais Brazil; 2 Department of Health Sciences, Universidade Federal de Lavras (UFLA), Mailbox 3037, Lavras, Minas Gerais 37200-900 Brazil; 3 Chemistry Department, Universidade Federal de Lavras, Mail Box 3037, Lavras, Minas Gerais Brazil; 4 Agriculture Department, Universidade Federal de Lavras, Mail Box 3037, Lavras, Minas Gerais Brazil; 5 Agrarian Sciences Institute, Universidade Federal dos Vales do Jequitinhonha e Mucuri, Unaí, Minas Gerais 38610-000 Brazil; 6 Department of Foundational Sciences, School of Dental Medicine, East Carolina University (ECU), Greenville, NC 27834 USA

**Keywords:** Plant extracts, Obesity, Toxicity, Pharmacology, Candlenut, Physiology

## Abstract

**Background:**

Candlenut (CN) has been used indiscriminately for weight loss. In vivo effects of CN in different doses are scarce.

**Objective:**

To evaluate the effects of CN ingestion in obese rats.

**Design:**

Thirty animals (obese and non-obese) received one of three different types of treatments: placebo, CN ingestion in a popular therapeutic regimen (8 days with oral administration of 0.2 mg/kg followed by 20 days with doses of 0.4 mg/kg), and ingestion of a doubled popular dose—called 2CN. Treatment was maintained for 28 days.

**Results:**

The fatty acid profile of CN indicated mainly linolelaidic and palmitoleic acids. Rats receiving CN and 2CN showed reduced plasmatic levels of glucose and lipoproteins (p < 0.05). A dose-dependent carcass fat reduction was observed (p < 0.05). Blood levels of aspartate aminotransferase (AST) and gamma-glutamyl transferase (GGT) reduced with CN and increased with 2CN doses (p < 0.05). Alanine aminotransferase (ALT) and the atherogenic index remained similar among all treatments (p > 0.05). Hepatic vacuolation decreased with CN, but the 2CN dose produced mononuclear leucocyte infiltrate.

**Conclusions:**

Although CN presented beneficial effects on the metabolism of rats, it also caused increased risk of liver damage.

## Introduction

Natural products and herbal medicines have been used indiscriminately for weight loss [1]. However, many of these compounds lack studies with scientific evidence of therapeutic potential or health risks [2]. The seed of *Aleurites moluccana (l.) Willd* (AM), also known as Kukui in Hawaii, candlenut (CN) in the US and Tuitui in the Cook Islands [3], has been used in the last years because of its medicinal properties.

The genus *Aleurites* is subdivided into *Aleurites montana, Aleurites trisperma, Aleurites cordata, Aleurites fordii* and *Aleurites moluccana*. The leafs of the plant *Aleurites moluccana* (AM) has been used to treat gastritis, fever, pain, diarrhea, asthma, and inflammation [4, 5] showing antibacterial [6] and antiviral effects [7]. AM’s bark dichloromethane extract contains acetyl aleuritolic acid, atraric acid, spruceanol, (5β,10α)-12-hydroxy-13-methoxy-8,11,13-podocarpatrien-3-one and sonderianol [8]. Besides, phytochemical composition of aqueous extract from AM seed present five mainly compounds: procyanidin dimer B1; 6-C-pentosyl-8-C-hexosyl apigenin; isovitexin; 6-C-pentosyl-8-C-pentosyl luteolin and neriifolin [9]. These compounds are known to present mostly antinociceptive and anti-inflammatory activity [8, 9]. On the other hand, the esters and saponins found in AM seeds can cause vomiting and diarrhea as adverse effects [10]. The seed of the plant has gained notoriety as a fast weight-loss agent, despite the scarcity of information about its pharmacological mechanisms of action. The supposed weight loss induced by the seed is attributed to laxative and diuretic properties. Some reports indicate diarrhea with fluid and electrolytes loss, mild to severe de-hydration and even death [11].

Sound in vivo studies proving its safety and efficacy are rare. Cases of adverse effects associated with the consumption of this seed in different parts of the world have been reported [11]. Several countries prohibited its trade, but illegal sales and consumption seems to persist, justifying the pre-clinical study of the effects of this seed. Thus, due to the scarcity of information on the in vivo effects of CN in different doses, the present study was aimed to analyze the effects of consuming this seed on obese and non-obese Wistar rats.

## Methods

### Animals

The experimental protocol was performed according to the Guide for the Use and Care of Laboratory Animals (ARRIVE guidelines for reporting in vivo experiments). The study was approved by the Ethics Committee on Animal Use of the Federal University of Lavras (UFLA) under protocol number CEUA 067/2016. Animals were treated in accordance with Guide for the Care and Use of Laboratory Animals (8th edition, National Academies Press). The sample size was determined to provide 80% power to recognize a significant difference of 20% among groups and a standard deviation of 15% with a 95% confidence for body weight (α = 0.05). Therefore, a sample size of five animals per group was required. Thirty adults male Wistar rats (*Rattus norvegicus albinus*) from the Central Animal Laboratory were used. The animals were healthy, with 90 days of age and an initial weight of 350 ± 28.7 g.

Firstly, we randomly distributed the animals into polypropylene cages (49 × 34 × 16 cm) and they underwent an acclimation period of 7 days under optimal conditions of temperature (22 ± 2 °C), humidity (45 ± 15%), and photoperiod (12/12 h light/dark cycles). Commercial feed and water were provided ad libitum. The commercial diet (Nuvilab CR-1®) contained 25.6% kcal protein, 62.6% kcal carbohydrate, 11.8% kcal lipid and 0.006% diet vitamin E.

**Fig. 1:**
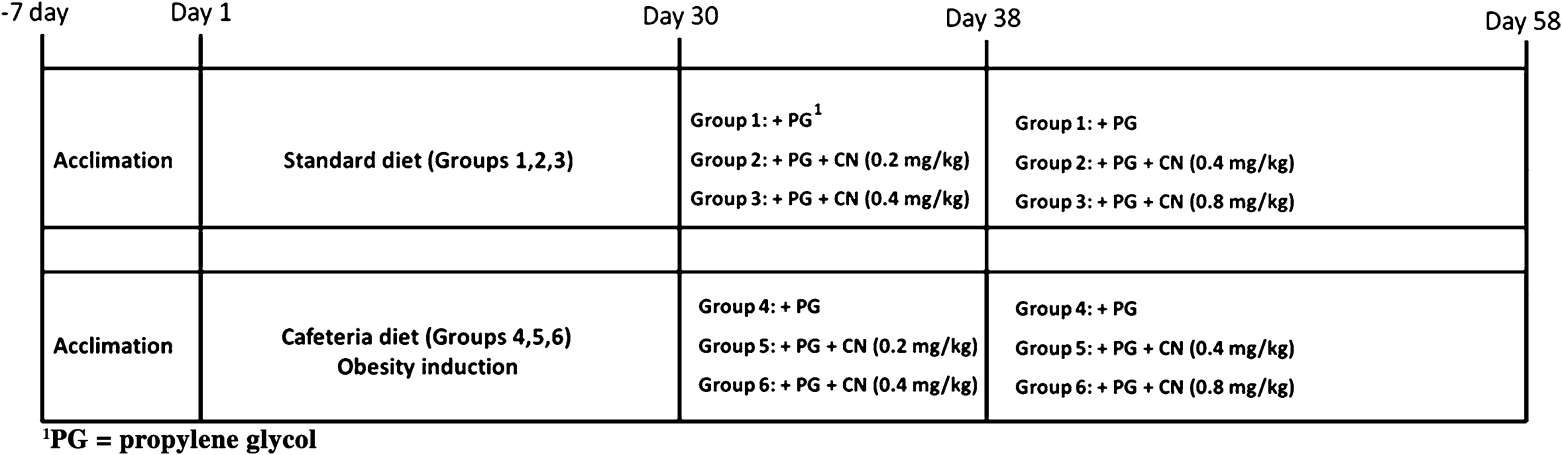
Schematic representation of the experimental design over time for animals receiving a standard diet (groups 1, 2 and 3) and cafeteria diet (groups 4, 5 and 6)

Half of animals received a commercial diet for 30 days. The other half was induced to obesity by receiving a hypercaloric diet according to Oliveira et al [12]. The diet consisted of commercial feed (37%), roasted peanuts (25%), milk chocolate (25%) and cornstarch cookies (13%) at a 3:2:2:1 ratio. These ingredients were ground, mixed and offered in the form of pellets [12]. After 30 days of obesity induction, for the subsequent 28 days, CN was administered. During this period, the animals continued to receive their respective diet (Table 1). The schematic representation of the experimental design over time is shown in Fig. 1.

**Table 1:** Characterization of the experimental groups of male Wistar rats according to their respective diet and candlenut treatments (n = 5 animals/group)

Groups	Diets
1	Standard diet + propylene glycol
2	Standard diet + propylene glycol + CN
3	Standard diet + propylene glycol + 2CN
4	Cafeteria diet + propylene glycol
5	Cafeteria diet + propylene glycol + CN
6	Cafeteria diet + propylene glycol + 2CN

CN: popular dose of candlenut (8 days with oral administration of 0.2 mg/kg followed by 20 days with oral doses of 0.4 mg/kg). 2CN: twice the popular dose of candlenut

Obesity was determined by the Lee index: [cubic root of body weight (grams)/naso-anal length (centimeters) × 1000]. Values above 0.3 indicate obese animals [13]. The index was calculated on days 0, 30 and 58 of the experiment.

### Administration of the candlenut

CN was administered by gavage using an endoscopic needle coupled to a 1-mL syringe. The seeds were macerated and homogenized in propylene glycol (PG), which was used as vehicle. Only the raw seed was used in the solution. The placebo groups received only propylene glycol (control).

The initial CN dose administered was calculated in proportion to the body weight of the animals, using as a reference the popular dose used for a person of 100 kg (kg). For human treatment with CN, it is recommended to ingest 1/8 of the seed in the first 8 days of treatment, and then 1/4 of the seed in the remaining days, independently of body weight [11]. For rats in the present experiment, the proportional dose was 0.2 mg/day for the first 8 days and 0.4 mg/day for the 20 subsequent days, respectively. Regarding the 2CN treatment, the groups received 0.4 mg/day in the first 8 days and 0.8 mg/day on the other days, representing the doubled dose.

### Body weight and blood collection analysis

During the experimental period, food consumption was measured daily, and the rats were weighed weekly. At the end of the experimental period (day 58), the animals were euthanized by cardiac puncture under anesthesia (Sodium thiopental, 50 mg/kg intraperitoneally) after fasting for 8 h. The concentrations of total cholesterol, high density lipoprotein cholesterol (HDL-c), triacylglycerols (TAG), glucose, aspartate aminotransferase (AST), alanine aminotransferase (ALT), and gamma-glutamyltransferase (GGT) were determined using commercial colorimetric/enzymatic kits (Labtest Diagnostica S/A®; Lagoa Santa, MG, Brazil). The low-density lipoprotein cholesterol (LDL-c) levels were calculated using the Friedewald formula, where LDL-c = Total Cholesterol − HDL-c − Triacyclglycerols/5. The very low-density lipoprotein cholesterol (VLDL-c) values were measured using the following equation: VLDL-c = Triacyclglycerols/5 [14]. The atherogenic index of plasma was calculated using the equation: log (TG)/(HDL-C), which is used as a significant predictor of atherosclerosis [15].

### Histopathological analysis

The liver was collected and fixed in 10% buffered formaldehyde and was subsequently dehydrated in an increasing alcohol series, cleared in xylol, and embedded in paraffin to obtain 5-μm-thick slices as described previously [16]. Subsequently, the sections were stained with hematoxylin–eosin and analyzed under optical microscopy (Olympus CX31; Olympus, Tokyo, Japan) by a veterinarian pathologist blind to the treatments.

The microscopic lesions of the liver were classified regarding presence of cytoplasmic vacuolation, inflammatory infiltrate, necrosis, and vessel congestion. Besides, the lesions were classified as: incipient, when there were lesions in individualized cells; focal, when it occurred in a single point; multifocal, when it occurred at various points sparsely; focally extensive, when a considerable area of the parenchyma was affected; and diffuse, when all the parenchyma was affected. Additionally, liver tissue ratings were assigned according to the presence and/or degree of mononuclear infiltration as follows: No change; light-+; discreet-++; moderate-+++; severe-++++.

### Carcass composition

Carcasses were analyzed after removing skin, tail, paws, head, and viscera. The whole body was ground and homogenized to determine humidity, protein, collagen, fat, and mineral matter content. Near infrared reflectance (NIR) spectrometry was used, using the FoodScan™ NIR spectrophotometer (FOSS, Hillerod, Denmark) [17].

### CN fatty acid extraction by gas chromatography

Macerated CN (10 g) was added to 80 mL of solvent in a flask, connected to a bulb condenser. The extraction process included the use of three solvents in order to determine the one with the best yield and extraction capacity. Extraction was conducted under reflux for 6 h from the time of boiling. Then, the sample was filtered and then rotaevaporated until solvent free [18].

CN humidity was determined by adding approximately 3 g of the pulp and 80 mL of cyclohexane in a round-bottomed flask, which was coupled to the Dean stark apparatus. The flask was heated and after 2 h, the volume of water present in the plant material was quantified [19]. The CN oil yield was calculated and expressed as the weight of oil divided by the weight of the material on a moisture-free basis (MFB).

Next, 100 mg of CN oil, 2 mL of hexane (HPLC grade) and 0.2 mL of 2 mol/LKOH methanolic solution were added to a test tube. Thereafter, the tube was vortexed for 30 s. After stirring, 3 mL of saturated sodium chloride solution was added to the tube. Subsequently, the tube was allowed to stand for phase separation. The upper layer was removed for chromatographic analysis [18, 20].

Fatty acid (FA) composition was determined on a gas chromatograph (model Shimadzu CG-17a). We used a SP 2560 column 100-mm long by 0.25 mm in internal diameter and 0.2 µm thickness of the liquid-phase film with a flame ionization detector at 260 °C, split injector (1:20 ratio) at 260 °C with an initial oven temperature of 140 °C for 5 min. As a qualitative analysis, the chromatogram obtained by the injection of the samples was compared with that obtained for the PUFA standard, with integration of the peaks. Fatty acids were identified by comparison of retention times.

### Statistical analysis

The study design was completely randomized with a 2 × 3 factorial arrangement (obese or non-obese and three treatments: without CN, CN and 2CN). The data were subjected to analysis of variance (two-way ANOVA), and the means were compared by Tukey’s test at 5%. The Lee index values along time were compared using three-way-ANOVA. The analyses were performed using the SAS statistical program (1996).

## Results

**Fig. 2:**
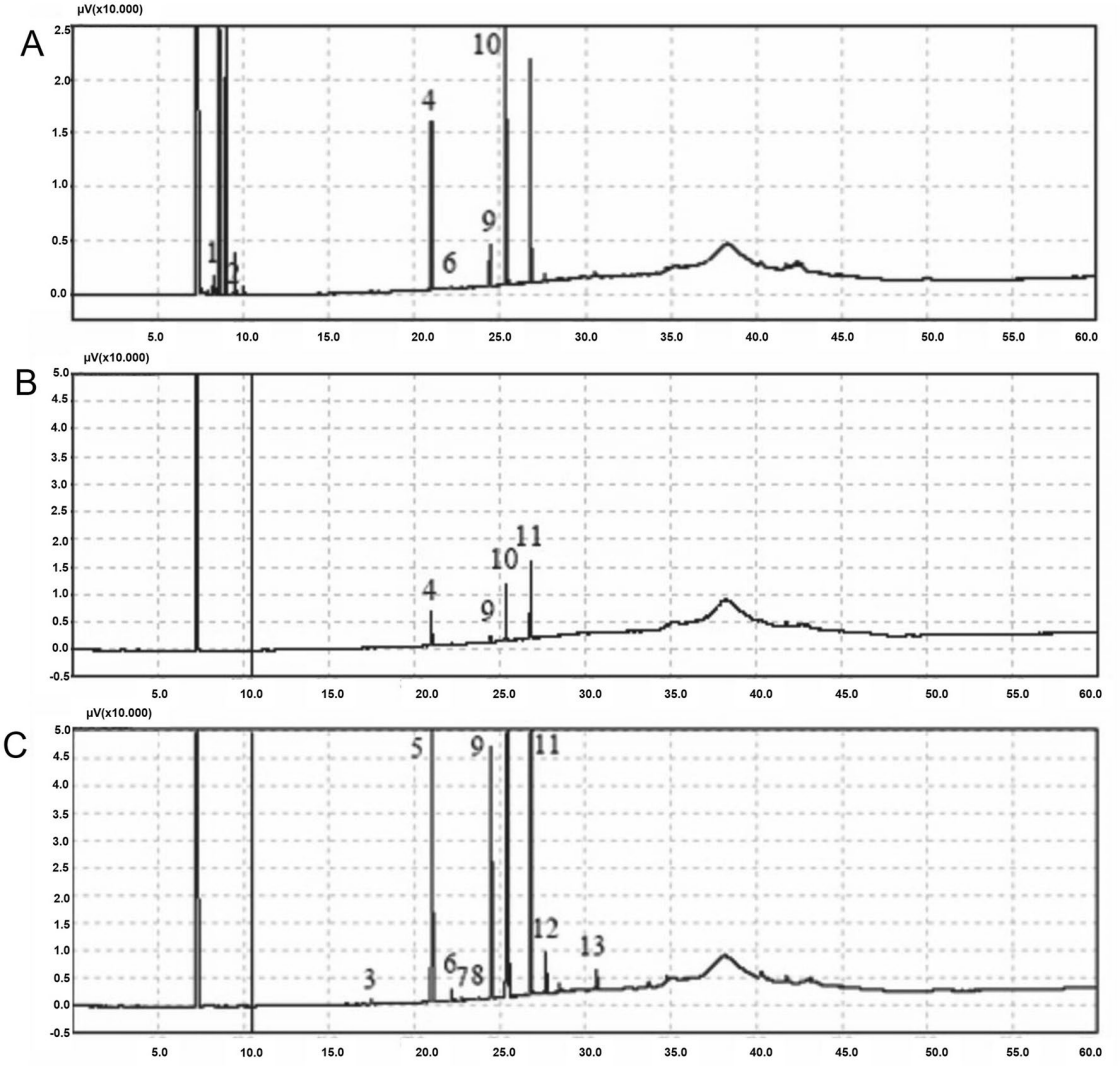
Chromatograms of the solvents (**A** hexane; **B** methanol; **C** methanol followed by centrifugation) used in the extraction of fatty acids from candlenuts seed

The major fatty acid found on candlenut oil were methyl palmitate, methyl palmitoleate, linolelaidic acid methyl ester and methyl arachidate. Besides, the trans-9-elaidic acid methyl ester was the only component found in all extraction protocols using three different solvents (Table 2; Fig. 2).

**Table 2:** Fatty acid chemical composition of candlenut oil according to solvents used in extraction

Number	Identified fatty acids	Retention time	Hexane	Methanol	Centrifuged methanol
			% of Area		
1	Methyl hexanoate	8.320	1.3904	–	–
2	Methyl octanoate	9.416	0.1678	–	–
3	Methyl myristate	17.457	–	–	0.1755
4	Methyl palmitate	20.998	21.2427	19.8421	–
5	Methyl palmitoleate	21.046	–	–	37.4686
6	Methyl heptadecanoate	22.176	0.3760	–	0.4484
7	Cis-10-heptadecenoic acid methyl ester	22.761	–	–	0.1612
8	Methyl stearate	23.789	–	–	0.085
9	Trans-9-elaidic acid methyl ester	24.496	5.9588	3.8231	10.3682
10	Linolelaidic acid metheyl ester	25.348	40.3870	33.6412	–
11	Methyl arachidate	26.842	–	41.6657	48.0404
12	Methyl eicosanoate	27.679	–	–	2.1375
13	Cis-11,14,17-eicosatrienoic acid methyl ester	30.657	–	–	1.1152

**Fig. 3:**
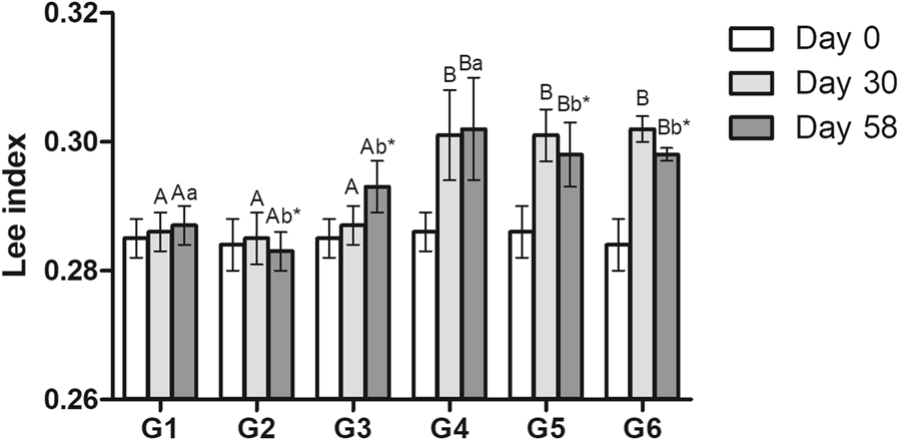
Lee index (Mean ± SD) of the of non-obese (G1, G2, G3) and obese (G4, G5 and G6) male Wistar rats on day 0 after induction of obesity with a cafeteria diet (day 30) and after 28 days under treatment by gavage with placebo (propylene glycol—G1 and G4) or CN (0.2 mg/kg/day CN for 8 days and 0.4 mg/kg/day for the subsequent 20 days—G2 and G5) or 2CN (0.4 mg/kg/day CN for 8 days and 0.8 mg/kg/day for the subsequent 20 days—G3 and G6). ^A,B^ Different letters indicate significant difference in relation to diet. ^a,b^ Different letters indicate significant difference in relation to the respective treatment. ^*^Indicate difference from days 30 and 58. Three-way ANOVA followed by Tukey's test (p < 0.05)

After 30 days receiving cafeteria-diet, all animals from groups 4 to 6 were metabolically obese (Lee index > 0.3). Lee index of Animals from groups 1 to 3 presented values < 0.3. After 28 days of CN treatment, the obese animals showed a significant reduction of the Lee index, reaching values under the obesity range (Fig. 3).

**Fig. 4:**
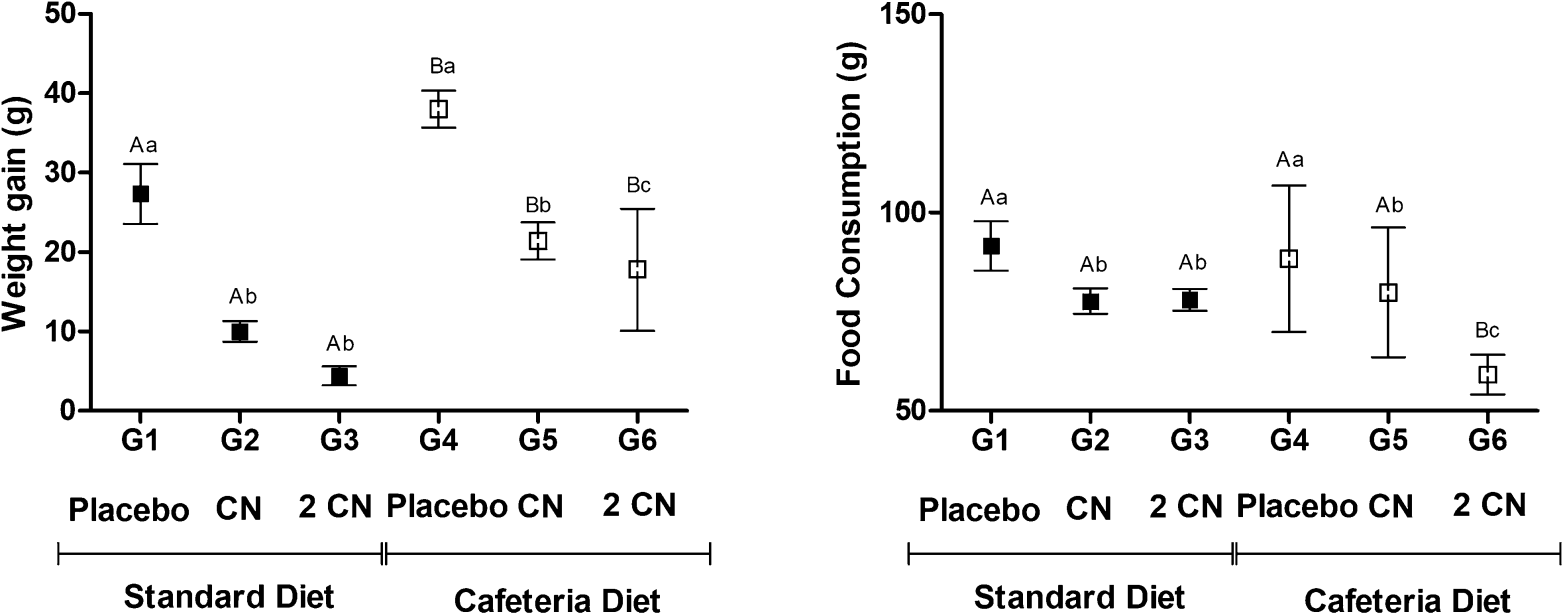
Weight gain and food consumption (Mean ± SD) of non-obese (G1, G2, G3) and obese (G4, G5 and G6) male Wistar rats for 28 days under treatment by gavage with placebo (G1 AND G4), CN (0.2 mg/kg/day CN for 8 days and 0.4 mg/kg/day for the subsequent 20 days—G2 and G5) or 2CN (0.4 mg/kg/day of CN for 8 days and 0.8 mg/kg/day for the subsequent 20 days—G3 and G6). ^A,B^ Different letters indicate significant difference in relation to the diets. ^a,b,c^ Different letters indicate significant difference in relation to the treatments. Two-way ANOVA followed by Tukey's test (p < 0.05)

Weight gain was higher in the animals that consumed the cafeteria diet. CN and 2CN ingestion significantly decreased weight gain for 28 days (p < 0.05) both in the cafeteria and the standard diet groups (Fig. 4). The amount of consumed feeding (in grams) was not different in groups receiving standard and cafeteria diets (p > 0.05), although cafeteria diet was hypercaloric. CN ingestion decreased food consumption also for both standard and cafeteria groups (p < 0.05), with a dose-dependent effect for the cafeteria group only (Fig. 4).

**Fig. 5:**
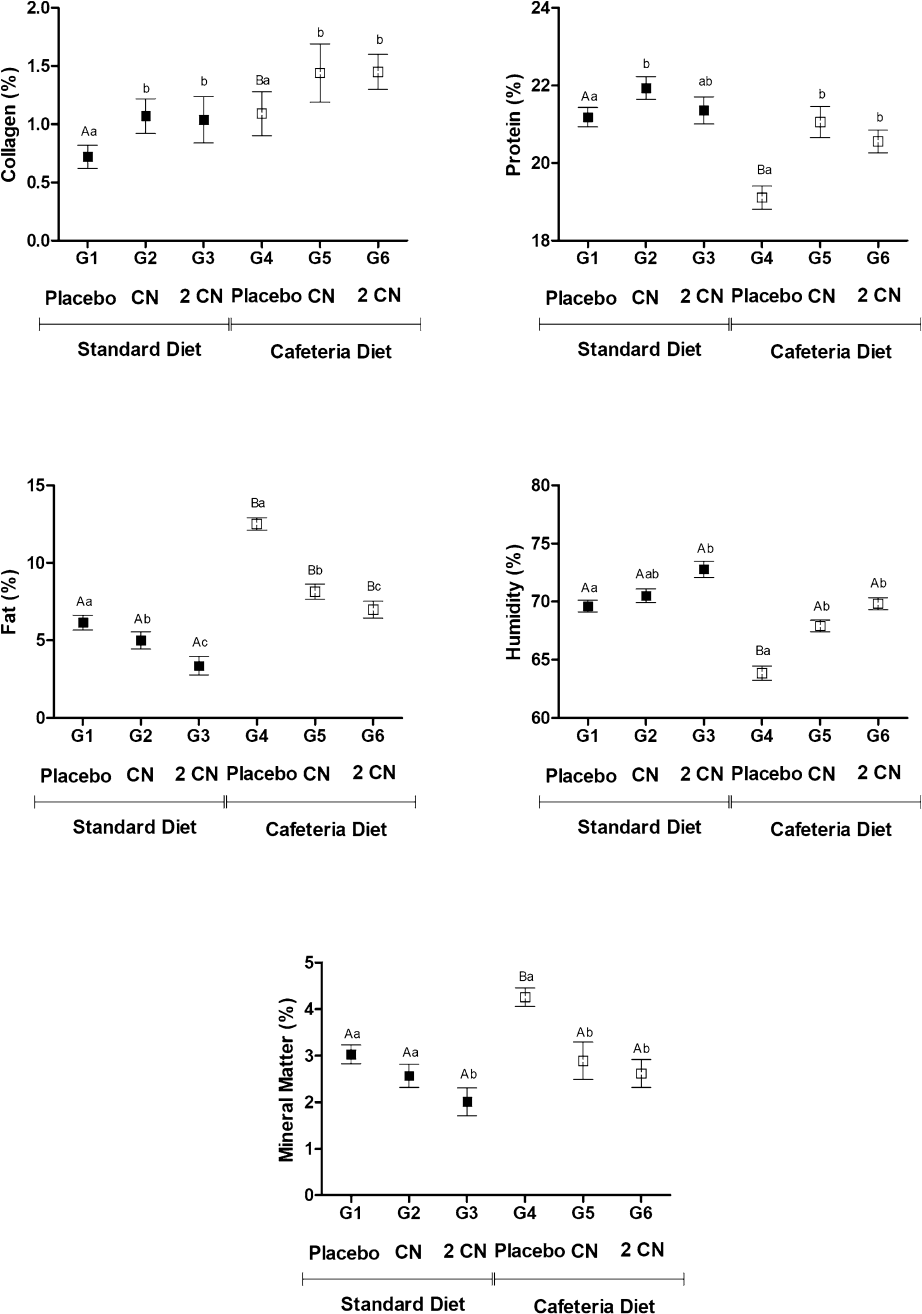
Chemical composition (Mean ± SD) of the carcass (%) of non-obese (G1, G2, G3) and obese (G4, G5 and G6) male Wistar rats after induction of obesity with a cafeteria diet (day 30) and after 28 days under treatment by gavage with placebo (propylene glycol—G1 and G4) or CN (0.2 mg/kg/day CN for 8 days and 0.4 mg/kg/day for the subsequent 20 days—G2 and G5) or 2CN (0.4 mg/kg/day CN for 8 days and 0.8 mg/kg/day for the subsequent 20 days—G3 and G6). ^A,B^ Difference in relation to the diets. ^a,b^ Difference in relation to the treatments (placebo, CN or 2CN). ANOVA followed by Tukey’s test (p < 0.05). SEM: standard error of the mean

Cafeteria-diet increased collagen, fat and mineral matter while decreased protein and humidity in carcass. CN ingestion increased collagen, humidity and protein content while reduced fat percentage in carcass (p < 0.05) (Fig. 5). Significant interaction existed be-tween diet and CN ingestion for fat and humidity parameters (p < 0.05).

**Fig. 6:**
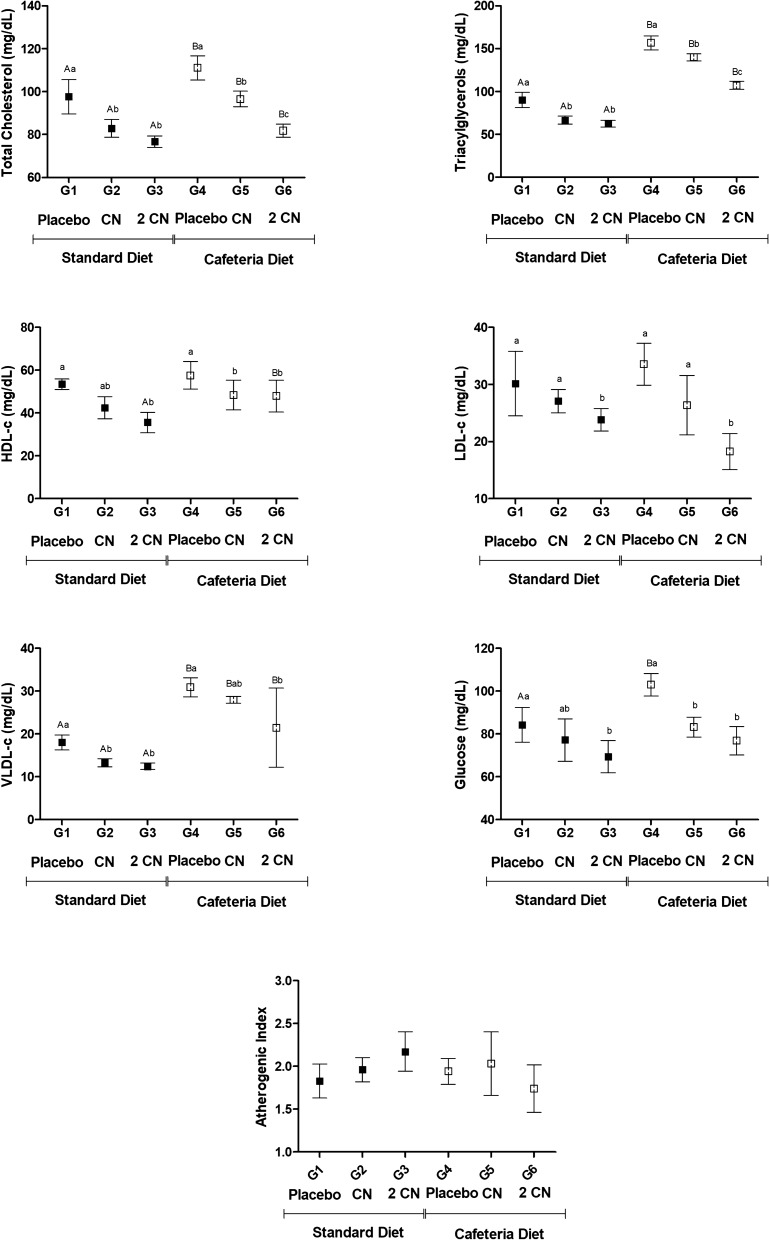
Serum biochemical parameters (plasma lipoprotein and glucose) and atherogenic index (Mean ± SD) in non-obese (G1, G2, G3) and obese (G4, G5 and G6) male Wistar rats after 28 days under treatment by gavage with placebo (propylene glycol—G1 and G4), CN (0.2 mg/kg/day CN for 8 days and 0.4 mg/kg/day for the subsequent 20 days—G2 and G5) or 2CN (0.4 mg/kg/day CN for 8 days and 0.8 mg/kg/day for the subsequent 20 days—G3 and G6). ^A,B^ Different letters indicate significant difference in relation to the diets. ^a,b,c^ Different letters indicate significant difference in relation to the treatments. Two-way ANOVA followed by Tukey's test (p < 0.05)

**Fig. 7:**
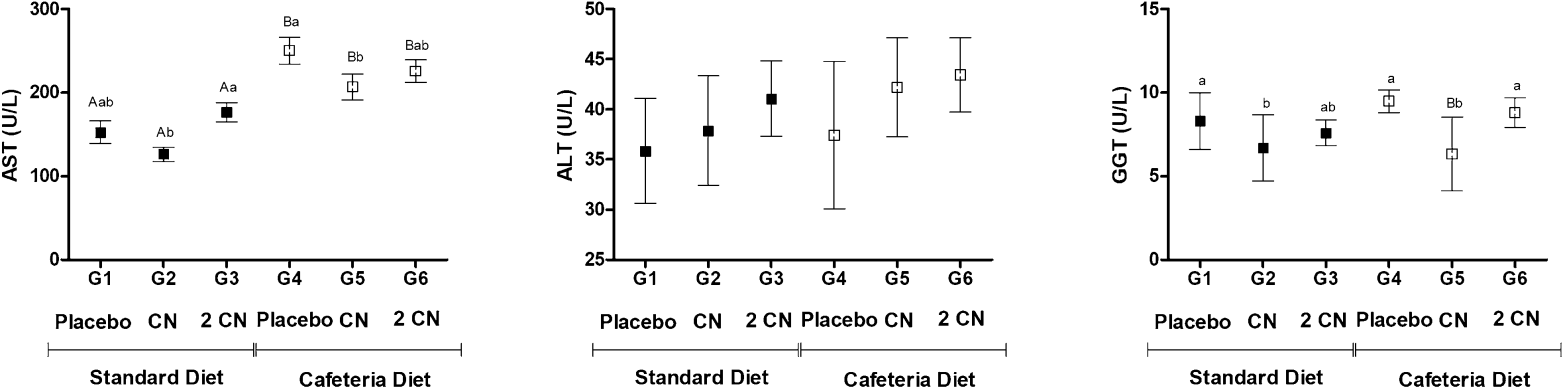
Plasma concentrations (Mean ± SD) of hepatic enzymes AST—aspartate aminotransferase (U/L); ALT—alanine aminotransferase (U/L) and GGT—gamma-glutamyltransferase (U/L of obese male (G4, G5 and G6) and non-obese Wistar rats after 28 days under treatment by gavage with placebo (propylene glycol—G1 and G4) or CN (0.2 mg/kg/day CN for 8 days and 0.4 mg/kg/day for the subsequent 20 days—G2 and G5) or 2CN (0.4 mg/kg/day CN for 8 days and 0.8 mg/kg/day for the subsequent 20 days—G3 and G6). ^A,B^ Different letters indicate significant difference in relation to the diets. ^a,b^ Different letters indicate significant difference in relation to the treatments. Two-way ANOVA followed by Tukey's test (p < 0.05)

Concerning plasma lipoproteins, the concentrations of triacylglycerols, total cholesterol and fractions were generally increased by cafeteria diet and reduced by CN (p < 0.05) (Fig. 6). Besides, we also found significant reductions in blood glucose levels (p < 0.05). Blood glucose levels of obese rats reaching values similar to the groups receiving standard diet (Fig. 6). Atherogenic index were similar between diets and among treatments (Fig. 6). Liver enzymes AST and GGT, decreased when the CN dose was administered. However, their values increased (p < 0.05) in groups receiving 2CN treatment (Fig. 7). ALT enzyme concentration was similar between diets and among treatments (Fig. 7).

**Fig. 8:**
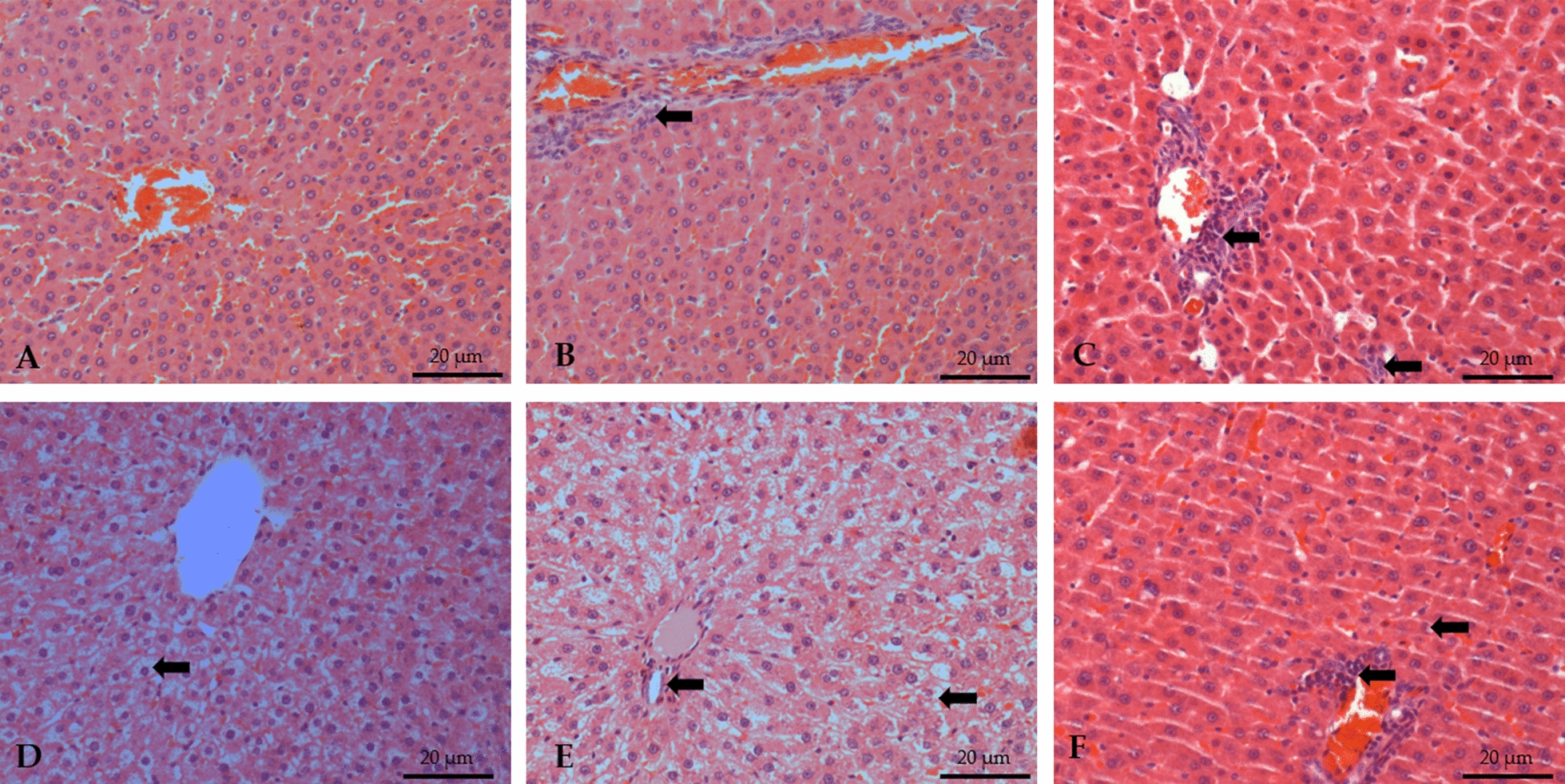
Photomicrograph (magnification ×20) of the hepatic parenchyma stained with hematoxylin–eosin (HE) of non-obese male (**A**–**C**) and obese (**D**–**F**) Wistar rats after 28 days under treatment by gavage with placebo (propylene glycol), CN (0.2 mg/kg/day CN for 8 days and 0.4 mg/kg/day for the subsequent 20 days) or 2CN (0.4 mg/kg/day CN for 8 days and 0.8 mg/kg/day for the subsequent 20 days). **A** G1: normal hepatic parenchyma. **B** G2: discrete mononuclear infiltrate in the portal space (arrow), without degenerative or necrotic lesions in hepatocytes. **C** G3: normal hepatocytes. **D** G4: hepatocytes with moderate diffuse cytoplasmic vacuolation (arrows). **E** G5: some hepatocytes with mild cytoplasmic vacuolation (arrows). **F** G6: mild mononuclear infiltrate (arrow) and rare hepatocytes with cytoplasmic vacuolation (HE)

Rats receiving cafeteria diet presented hepatic vacuolation, and the degree of degeneration decreased slightly with CN and 2CN treatments. However, a mononuclear infiltrate was found in the liver of animals that consumed candlenut, especially 2CN. Ballooning and fibrosis were not reported in the histopathological evaluation of the present study (Fig. 8). Both placebo groups (cafeteria and control diets) presented discrete to mild (+/++) mononuclear infiltrate, while CN and 2CN groups had moderate to severe in-filtrates (+++ /++++).

## Discussion

The results of the present study demonstrated a reduction in weight gain and potential improvement of blood biochemical parameters of obese (and non-obese) rats that consumed candlenut for 28 days. In general, the effects were positive, with the reduction of the carcass fat; total cholesterol and its fractions; and also, a reduction of blood glucose levels. However, some negative effects were observed, such as HDL-c reduction and in-creased AST and GGT levels especially when CN dose was doubled.

In the present study, obesity was induced in Wistar rats using the cafeteria diet. At the end of 30 days, induced obesity was confirmed by the Lee index [13]. The said model is efficient in a short time [21]. The cafeteria diet is composed of more palatable foods that trigger hyperphagia, leading to increased body weight, hyper-adiposity and hyperglycemia [22], as observed in the placebo group (G4). Additionally, high consumption of sucrose and fat stimulates the release of dopamine, reducing short-term stress, which may also contribute to body weight gain [23].

Animals that ingested candlenut had a reduction in body weight, taking Lee index values below 0.3 after 28 days of treatment. This fact demonstrated that candlenut was able to reduce obesity and control weight gain in rats. Similar properties were found in a study carried out by Pedrosa et al., [24] using *Aleurites moluccana* leaf extracts. AM seed presents highest concentrations of phenolic compounds, tannins and flavonoids [9] which are phytochemicals with recognized effect to promote weight loss [25–27]. Furthermore, the presence of α- and β-amyrenone in the AM seed is associated with the inhibition of enzymes responsible for lipid and carbohydrate absorption [28, 29]. The mechanism of action of CN derivatives remains unclear. The loss of fluids and electrolytes is likely caused by its laxative and diuretic effects, and the report of diarrhea is common [11]. In the present study, diarrhea was not observed. However, such findings should be evaluated with caution because the animals were kept under controlled conditions, without interference of physical activity, other drugs, alcoholic beverages, anabolic substances, among other factors, hindering direct extrapolations to humans.

The carcasses of rats that consumed candlenut presented lower concentrations of fat and dry matter, which were concentration dependent—i.e., at the higher dose (2CN), both the animals feeding on the standard diet and cafeteria diet showed more intense reduction (p < 0.05). Such results confirm the ability of the seed to affect body fat accumulation in rats. Besides, these results may be related to the fatty acids present in the candlenut structure, especially linolelaidic (ω-6) and palmitoleic acids (ω-7). Linolelaidic acid acts as an intra-cellular signal to induce the transcription of genes involved in lipid oxidation and inhibits the expression of genes involved in lipogenesis [30] by binding to the PPAR-α nuclear receptors (peroxisome proliferator-activated receptors) [31–34]. Additionally, it acts by inhibiting cholesterol biosynthesis by inhibiting the actions of SREBP-1 and SREBP-2 (sterol-regulatory element-binding proteins) [35]. Furthermore, palmitoleic acid acts as an adipokine, a signaling molecule produced by adipocytes, stimulating the effects of insulin on the muscles and reducing hepatic steatosis [36]. These mechanisms can also explain the effects of CN in reducing total cholesterol and LDL-c, HDL-c, VLDL-c fractions, and triglycerides.

In obese animals, it was possible to observe that the 2CN dose reduced LDL-c (p < 0.05), without significantly altering HDL-c. These results differ from those found previously [24] for *Aleurites moluccana* leaves, who found a reduction of LDL-c values and an increase in HDL-c levels. The different behaviors of LDL-c and HDL-c parameters in response to candlenut indicate a variation in the distribution of cholesterol lipoproteins, perhaps with increased hepatic receptors that catabolize LDL-c [37]. Indeed, our results demonstrated the hypocholesterolemic properties of CN. Reduced LDL-c levels are associated to a reduced atherosclerosis risk [38]. The reduction in HDL-c levels is not a desirable outcome, as this lipoprotein is related to lower cholesterol deposition, and consequently, less chance of atherosclerotic plaque formation in the vascular endothelium [39].

In this study, blood glucose levels were also significantly reduced in both obese and non-obese animals from groups that received CN. Hyperglycemia is commonly found in obese individuals and represent a sign of insulin resistance [40]. However, the reduction in glycemia observed in the present study in non-obese normoglycemic animals highlights another undesirable effect of CN, since the lower availability of circulating glucose affects the proper functioning of several organ systems. Thus, hypocholesterolemia caused by CN associated to blood glucose decrease, may be related to alterations in the intestinal absorption of nutrients, compromising the availability of circulating energy.

Regarding the liver enzymes AST and GGT, there was a reduction of their plasma concentrations in the obese rats that received the low CN dose, but the AST values in-creased with the 2CN dose—even when compared with both non-obese and obese placebo groups. Increased levels of AST may be related to liver damage or disease [41]. In addition, this outcome may be possibly due to hepatoxicity induced by high doses of CN. Higher transaminases levels are associated with hepatic toxic events such as cell membrane disruption, mitochondrial dysfunction, oxidative stress, and recruited inflammatory cells [42]. To corroborate the suspicion of liver damage, we observed hepatic mononuclear infiltrate appeared in the samples from animals that ingested the seed at the different doses, indicating the onset of hepatic inflammation. Although liver damage observed on AST may be considered small, it appeared after only a short period of 28 days of CN treatment. We decided to evaluate 28 days therapy due to the normal popular consumption for this medicinal plant. However, abuse is not uncommon and deserves proper investigation. Future studies should evaluate chronic consumption of CN for longer periods.

Regarding the liver enzymes AST and GGT, there was a reduction of their plasma concentrations in the obese rats that received the CN dose (p < 0.05), but the GGT values in-creased with the 2CN dose (p < 0.05). Additionally, rats consuming a cafeteria diet had hepatic vacuolation, which was reduced slightly with the CN and 2CN treatments, probably due to the reduction in body fat caused by the seed. Hepatocyte vacuolation is associated with non-alcohol-related fatty liver disease [43]. These results demonstrate that consumption of CN attenuated the accumulation of lipids in the liver. However, a hepatic mononuclear infiltrate appeared in the samples from animals that ingested the seed at the different doses, indicating the onset of hepatic inflammation. There is a shortage of data on the toxicity of *Euphorbiaceae* species in rats and humans, but these species are potentially toxic, as observed in other species [44]. One should consider that humans often associate candlenut ingestion with food restriction, overtraining, other drugs, alcoholic beverages, anabolic substances, which can overload liver function leading to higher probability of adverse effects. Studies at higher doses or for longer periods are necessary to assess the potential toxicity of the seed.

In summary, candlenut presented favorable results regarding weight control, body fat, blood lipoproteins and glucose levels. However, it is of paramount importance to note that these effects were achieved in Wistar rats under controlled laboratory conditions. Future studies are encouraged in order to elucidate toxicological aspects elicited in the pre-sent study. In humans, the combination of natural products, excess physical activity and/or other drugs is common, and the potential associated effects are still unknown. Additionally, the seed has been used by the population worldwide, with reports of toxicity and death [11]. Indiscriminate use of candlenut, whether at high amounts or for a pro-longed time, is reported to cause feelings of discomfort and nausea. These symptoms may be followed by vomiting, abdominal pain, diarrhea, dehydration and changes in the heart rate. Future studies analyzing the effects of the consumption of the seed on the blood pressure and molecular pathways are necessary to ensure its safe use. Additionally, it is interesting that future studies evaluate the effects of CN consumption on hormones involved with food ingestion control, such as leptin.

In conclusion, CN consumption for 28 days reduced body weight, carcass fat, plasma lipoproteins, blood glucose levels and the degree of vacuolation in Wistar rats. Higher doses were associated to mononuclear liver infiltrate and increased AST and GGT levels, indicating the risk of liver overload and damage.

## Data Availability

The datasets used and/or analyzed during the current study are available from the corresponding author on reasonable request.

## References

[1] Cercato LM, White PAS, Nampo FK, Santos MRV, Camargo EA (2015). A systematic review of medicinal plants used for weight loss in Brazil: is there potential for obesity treatment?. J Ethnopharmacol.

[2] Neergheen-Bhujun VS (2013). Underestimating the toxicological challenges associated with the use of herbal medicinal products in developing countries. Biomed Res Int.

[3] Cesca TG, Faqueti LG, Rocha LW, Meira NA, Meyre-Silva C, De Souza MM (2012). Antinociceptive, anti-inflammatory and wound healing features in animal models treated with a semisolid herbal medicine based on *Aleurites moluccana* L. Willd. Euforbiaceae standardized leaf extract: Semisolid herbal. J Ethnopharmacol.

[4] Mendes Hoepers S, Tolentino De Souza HGM, Meira Quintão NL, Roberto Santin J, Cechinel Filho V, Silva RML (2015). Topical anti-inflammatory activity of semisolid containing standardized *Aleurites moluccana* L. Willd (Euphorbiaceae) leaves extract. J Ethnopharmacol.

[5] Quintão NLM, Antonialli CS, Da Silva GF, Rocha LW, De Souza MM, Malheiros A (2012). *Aleurites moluccana* and its main active ingredient, the flavonoid 2″-O-rhamnosylswertisin, have promising antinociceptive effects in experimental models of hypersensitivity in mice. Pharmacol Biochem Behav.

[6] Locher CP, Burch MT, Mower HF, Berestecky J, Davis H, Van Poel B (1995). Anti-microbial activity and anti-complement activity of extracts obtained from selected Hawaiian medicinal plants. J Ethnopharmacol.

[7] Locher CP, Witvrouw M, De Bethune MP, Burch MT, Mower HF, Davis H (1996). Antiviral activity of Hawaiian medicinal plants against human immunodeficiency virus type-1 (HIV-1). Phytomedicine.

[8] De Souza MM, Chagas LGRD, Gonçalves AE, Tomczak M, Reichert S, Schuquel ITA (2021). Phytochemical analysis and antinociceptive properties of hydroalcoholic extracts of *Aleurites moluccanus* bark. Planta Med.

[9] Fukuda de Castilho P, Gomes da Silva Dantas F, Pires de Araújo R, Almeida Castro LH, Souza de Araújo FH, Negri M (2021). General and genetic toxicology studies of *Aleurites moluccana* (L.) Willd. seeds in vitro and in vivo assays. J Ethnopharmacol.

[10] Corcoran J, Gray T, Bangh SA, Singh V, Cole JB (2020). Fatal yellow oleander poisoning masquerading as benign candlenut ingestion taken for weight loss. J Emerg Med.

[11] González-Stuart AH, Rivera JO (2017). Toxicity of candlenut seed (*Aleurites moluccanus*), a purported herbal weight loss supplement. Pharmacologia.

[12] Oliveira TWS, Leandro CG, De Jesus Deiró TCB, Dos Santos PG, Da França SD, Druzian JI (2011). A perinatal palatable high-fat diet increases food intake and promotes hypercholesterolemia in adult rats. Lipids.

[13] Bernardis LL, Patterson BD (1968). Correlation between “Lee index” and carcass fat content in weanling and adult female rats with hypothalamic lesions. J Endocrinol.

[14] Sposito AC, Caramelli B, Fonseca FAH, Bertolami MC, Rassi A, Neto AA (2007). IV Diretriz Brasileira sobre dislipidemias e prevenção da aterosclerose Departamento de Aterosclerose da Sociedade Brasileira de Cardiologia. Arq Bras Cardiol.

[15] Hemmati M, Zohoori E, Mehrpour O, Karamian M, Asghari S, Zarban A (2015). Anti-atherogenic potential of jujube, saffron and barberry: anti-diabetic and antioxidant actions. EXCLI J.

[16] Andrade EF, Lobato RV, Araújo TV, Orlando DR, Gomes NF, Alvarenga RR (2014). Metabolic effects of glycerol supplementation and aerobic physical training on Wistar rats. Can J Physiol Pharmacol.

[17] Gondim P, Rosa P, Okamura D, Silva V, Andrade E, Biihrer D (2018). Benefits of fish oil consumption over other sources of lipids on metabolic parameters in obese rats. Nutrients.

[18] AOAC (Association of Official Analytical Chemistry). Official Methods of Analysis. 17th ed. Chemists. A of OA, editor. Washington D. C.; 2000.

[19] Pimentel FA, Cardoso MDG, Salgado APSP, Aguiar PM, Silva VDF, De Morais AR (2006). A convenient method for the determination of moisture in aromatic plants. Quim Nova.

[20] Instituto Adolfo Lutz. Métodos físico-químicos para análise de alimentos - Secretaria da Saúde - Governo do Estado de São Paulo. 4^a^. Saúde S de E da, editor. São Paulo; 2008.

[21] Kretschmer BD, Schelling P, Beier N, Liebscher C, Treutel S, Krüger N (2005). Modulatory role of food, feeding regime and physical exercise on body weight and insulin resistance. Life Sci.

[22] South T, Westbrook F, Morris MJ (2012). Neurological and stress related effects of shifting obese rats from a palatable diet to chow and lean rats from chow to a palatable diet. Physiol Behav.

[23] Liang NC, Hajnal A, Norgren R (2006). Sham feeding corn oil increases accumbens dopamine in the rat. Am J Physiol - Regul Integr Comp Physiol.

[24] Pedrosa RC, Meyre-Silva C, Cechinel-Filho V, Benassi JC, Oliveira LFS, Zancanaro V (2002). Hypolipidaemic activity of methanol extract of *Aleurites moluccana*. Phyther Res.

[25] Boccellino M, D’Angelo S (2020). Anti-obesity effects of polyphenol intake: current status and future possibilities. Int J Mol Sci.

[26] Manzoor F, Un Nisa M, Hussain HA, Anwar H, Ahmad N, Umbreen H (2021). Therapeutic potential of hydrolysable tannin on weight management oxidative stress and reproductive health in polycystic rats. Food Sci Technol.

[27] Sandoval V, Sanz-Lamora H, Arias G, Marrero PF, Haro D, Relat J (2020). Metabolic impact of flavonoids consumption in obesity: from central to peripheral. Nutrients.

[28] Ferreira RGS, Silva WF, Veiga VF, Lima ÁAN, Lima ES (2017). Physicochemical characterization and biological activities of the triterpenic mixture α, β-amyrenone. Molecules.

[29] Hakim A, Jamaluddin J, Wahidah S, Idrus A, Wahab Jufri A, Nila B (2022). Ethnopharmacology, phytochemistry, and biological activity review of *Aleurites moluccana*. J Appl Pharm Sci.

[30] Xu J, Nakamura MT, Cho HP, Clarke SD (1999). Sterol regulatory element binding protein-1 expression is suppressed by dietary polyunsaturated fatty acids. A mechanism for the coordinate suppression of lipogenic genes by polyunsaturated fats. J Biol Chem.

[31] Ren B, Thelen AP, Peters JM, Gonzalez FJ, Jump DB (1997). Polyunsaturated fatty acid suppression of hepatic fatty acid synthase and S14 gene expression does not require peroxisome proliferator-activated receptor α. J Biol Chem.

[32] Nakatani T, Kim HJ, Kaburagi Y, Yasuda K, Ezaki O (2003). A low fish oil inhibits SREBP-1 proteolytic cascade, while a high-fish-oil feeding decreases SREBP-1 mRNA in mice liver: relationship to anti-obesity. J Lipid Res.

[33] Hashimoto Y, Yamada K, Tsushima H, Miyazawa D, Mori M, Nishio K (2013). Three dissimilar high fat diets differentially regulate lipid and glucose metabolism in obesity-resistant Slc: Wistar/ST rats. Lipids.

[34] Gao Q, Jia Y, Yang G, Zhang X, Boddu PC, Petersen B (2015). PPARα-deficient ob/ob obese mice become more obese and manifest severe hepatic steatosis due to decreased fatty acid oxidation. Am J Pathol.

[35] Deng X, Dong Q, Bridges D, Raghow R, Park EA, Elam MB (2015). Docosahexaenoic acid inhibits proteolytic processing of sterol regulatory element-binding protein-1c (SREBP-1c) via activation of AMP-activated kinase. Biochim Biophys Acta - Mol Cell Biol Lipids.

[36] Cao H, Gerhold K, Mayers JR, Wiest MM, Watkins SM, Hotamisligil GS (2008). Identification of a lipokine, a lipid hormone linking adipose tissue to systemic metabolism. Cell.

[37] Krause BR, Hartman AD (1984). Adipose tissue and cholesterol metabolism. J Lipid Res.

[38] Nunes SOV, Piccoli De Melo LG, Pizzo De Castro MR, Barbosa DS, Vargas HO, Berk M (2015). Atherogenic index of plasma and atherogenic coefficient are increased in major depression and bipolar disorder, especially when comorbid with tobacco use disorder. J Affect Disord.

[39] Di BBA, Psaltis PJ, Bursill CA, Nicholls SJ (2018). Translating evidence of HDL and plaque regression. Arterioscler Thromb Vasc Biol.

[40] Pirie FJ, Maharaj S, Esterhuizen TM, Paruk IM, Motala AA (2014). Retinopathy in subjects with type 2 diabetes at a tertiary diabetes clinic in Durban, South Africa: clinical, biochemical and genetic factors. J Clin Transl Endocrinol.

[41] Yakubu N, Oboh G, Olalekan A (2013). Antioxidant and hepatoprotective properties of tofu (Curdle Soymilk) against acetaminophen-induced liver damage in rats. Biotechnol Res Int.

[42] Pinafo M, Benedetti P, Gaiotte L, Costa F, Schoffen J, Fernandes G (2019). Effects of *Bauhinia forficata* on glycaemia, lipid profile, hepatic glycogen content and oxidative stress in rats exposed to Bisphenol A. Toxicol Reports.

[43] Aravinthan A, Verma S, Coleman N, Davies S, Allison M, Alexander G (2012). Vacuolation in hepatocyte nuclei is a marker of senescence. J Clin Pathol.

[44] Oliveira RB, Gimenez VMM, de Godoy SAP (2007). Intoxicações com espécies da família Euphorbiaceae. Rev Bras Biociências.

